# What Do Patients Consider to Be the Most Important Outcomes for Effectiveness Studies on Migraine Treatment? Results of a Delphi Study

**DOI:** 10.1371/journal.pone.0098933

**Published:** 2014-06-16

**Authors:** Antonia F. H. Smelt, Mark A. Louter, Dennis A. Kies, Jeanet W. Blom, Gisela M. Terwindt, Geert J. M. G. van der Heijden, Véronique De Gucht, Michel D. Ferrari, Willem J. J. Assendelft

**Affiliations:** 1 Department of Public Health and Primary Care, Leiden University Medical Center, Leiden, The Netherlands; 2 Department of Neurology, Leiden University Medical Center, Leiden, The Netherlands; 3 Department of Psychiatry, Leiden University Medical Center, Leiden, The Netherlands; 4 Department of Social Dentistry, Academic Center of Dentistry Amsterdam, VU Amsterdam University and University of Amsterdam, Amsterdam, The Netherlands; 5 Department of Psychology, Leiden University, Leiden, The Netherlands; 6 Department of Primary and Community Care, Radboud University Nijmegen Medical Centre, Nijmegen, The Netherlands; Harvard Medical School, United States of America

## Abstract

**Background:**

The outcome measures most frequently used in studies on the effectiveness of migraine treatment are whether the patient is free of pain, nausea, and free of photophobia/phonophobia within two hours. However, no patient-centred outcome measures are available. Therefore, we performed an online Delphi procedure to compile a list of outcome measures deemed most important to migraine patients.

**Methods:**

From a large database of migraine patients, we randomly selected 150 males and 150 females patients. We asked the open-ended question: ‘If a new medicine was developed for migraine attacks, what would you wish the effect of this medication to be?’ In the second and third rounds, we presented the answers of the first round and asked the patients to rate the importance of each item.

**Results:**

The initial response rate was 56% (n = 169). In the subsequent rounds the response rates were 90% (n = 152), and 97% (n = 147), respectively. Patients wanted their attack medication to treat the headache within 30 min, to prevent the attack from getting worse, to ensure they could function properly within 1 h, and prevent the recurrence of symptoms during the same day.

**Conclusions:**

The currently used outcome measures in migraine research do not sufficiently reflect the wishes of patients. Patients want the medication to work faster, to take away pain at an earlier stage, to make them able to function properly quickly, and to prevent recurrence. These aspects should be considered in future evaluation of new attack medication for migraine.

## Introduction

The most important outcome measure used in studies on the effectiveness of migraine treatment is whether the patient is pain free within two hours after taking the medicine [1]. Other symptoms assessed in this evaluation are nausea/vomiting and photophobia and phonophobia. The choice for these outcome measures is based on consensus among migraine specialists [1]. Despite claims that these outcome measures reflect the expectations of migraine patients, patients' wishes have only been explored by asking their opinion about the currently used outcome measures [2], [3]. To our knowledge, migraine patients have not been asked to add what they consider important themselves. Therefore, it can be questioned whether the currently used outcome measures in migraine research sufficiently reflect what is most relevant to the patients [4].

The importance of outcome measures relevant to patients was the rationale to start a Delphi study. The Delphi consensus method is commonly used within the health and social sciences to determine to what extent people agree about a given issue, or to transform opinion into group consensus. It is an iterative multistage process with a flexible approach to data collection most often in a series of structured questionnaires (rounds). In our study, the ‘experts’ (participants) anonymously completed the questionnaires in three rounds. The initial questionnaire collected qualitative comments, which were reported back to the participants in the second round in a quantative form. After the second round, the responses were summarized and reported to the participants in the third round [5], [6] This method has previously been used in the development of outcome measures [7].

In the present study we asked migraine patients to formulate their own outcome measures, with the aim to compile a short list of outcome measures that they considered most important. Also, we aimed to establish to what extent patients agree with the commonly used outcome measures. A similar project in patients with rheumatoid arthritis led to surprising results and the development of new outcome measures, that are now recommended in drug trials worldwide [8].

## Methods

We performed a Delphi procedure with web-based questionnaires that allowed patients to give their input over three rounds. In the first round we made an inventory of all possible opinions and we compiled a list of candidate items. In the second and third rounds we asked patients to evaluate these items.

### Patient panel

For this Delphi project, we randomly selected 150 male and 150 female patients from the Leiden University Medical center Neuro Analysis (LUMINA) database. We stratified patients for sex and treatment location (primary care or secondary care) in order to be able to detect differences between these groups of patients after answering the questions.

The LUMINA database includes over 54,000 adult migraine patients [9]. Of all the patients in this database, 87% has been diagnosed as migraine patient by a physician and 13% were self-reported migraineurs Of all patients 70% uses triptans. Upon entering the cohort, patients have to fill in an extensive questionnaire. In addition to questions necessary to accurately diagnose migraine, the questionnaire also includes items on demographic factors, acute and prophylactic headache medication use, and migraine attack frequency. Migraine diagnoses are established using a validated questionnaire based on the International Classification of Headache Disorders (ICHD-III) [10].

### Ethics statement

The LUMINA project has been approved by the Medical Ethics Committee of the LUMC. All participants of the LUMINA study provided written informed consent.

### Delphi questionnaires

**Figure 1:**
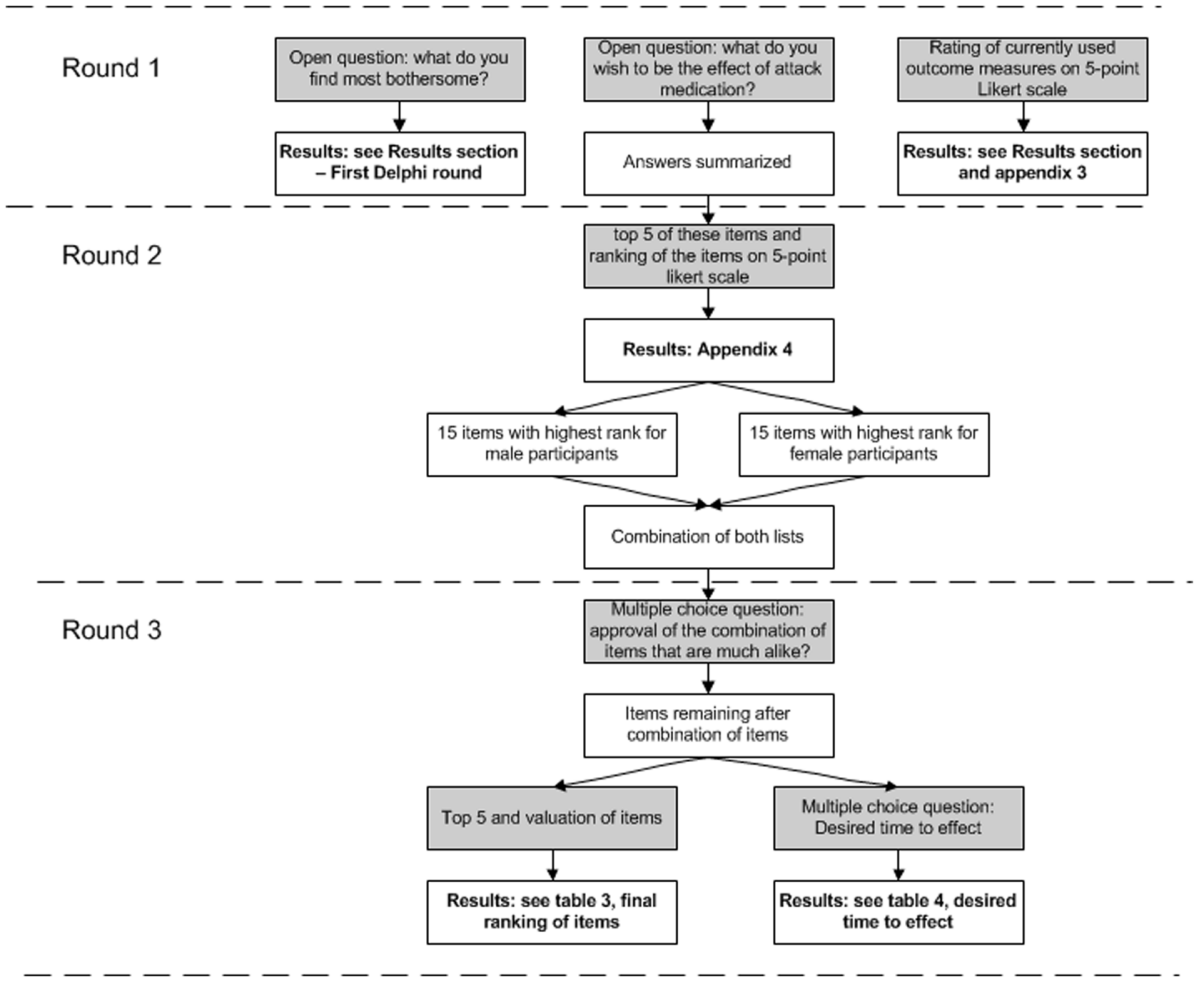
Contents of the three consecutive Delphi questionnaires. Grey boxes: contents of questionnaires. White boxes: actions by researchers.

Patients were sent an invitation by email to fill in three web-based questionnaires during a 6-month period. Figure 1 presents the questions asked in the consecutive rounds. The exact content of the questionnaires can be found in Appendix S1.

#### Round 1

First, we asked patients to provide information about their current headache status: number of migraine attacks per month, duration of migraine attacks, number of headache days per month, recurrence, and medication use.

Second, we posed two open-ended questions:

1. What do you find most bothersome about having a migraine attack?
2. If a new medicine was developed against migraine attacks, what would you wish the effect of this medication to be?

Patients were asked to list a minimum of three and a maximum of five answers, and to rate these answers on a 5-point Likert scale (from 1 = not important, to 5 = very important).

We then grouped the answers according to the presence of strong similarity. During this process, we followed an inductive method, i.e. answers were examined and those considered to be more or less the same were grouped as one item. No fixed number of items was set beforehand, in order to accommodate all new opinions. The answers were grouped by two of the authors (AS and VdG) separately, to ensure independence of assessments. Any discrepancies were resolved through a discussion with two other authors (ML and DK), who also checked whether they agreed with the items as formulated by AS and VdG.

Third, we asked patients to rate the relevance of the outcome measures currently used in clinical trials on a 5-point Likert scale (from 1 = not important, to 5 = very important). We extracted these outcome measures from the most recent guideline for controlled migraine drug trials and from a recently published questionnaire on the evaluation of migraine treatment [1], [11]. Patients were asked not to rate a listed outcome measure if they had not experienced it themselves.

The three questions in Round 1 were presented one by one, without the possibility to look back and change answers to the earlier questions. Thus, patients answered the open-ended questions (exploration of patients' opinions) without knowledge of the currently used outcome measures that were mentioned in the last step (existing criteria). In this way we ensured that participants were not informed about the content of the currently used outcome measures when answering the open-ended question.

#### Round 2

In the Round 2 we presented to the patients the categorized answers to the open-ended question ‘If a new medicine was developed against migraine attacks, what would you wish the effect of this medication to be?’ and asked to choose the five most important items and evaluate these on a 5-point Likert scale (from 1 = not important, to 5 = very important). The respondents were encouraged to comment on the list of items presented to them and to add any items that they felt had been left out.

Items from Round 2 were ranked according to the weight-frequency product, that was calculated based on the returned questionnaires, by multiplying the number of times an item was suggested byits mean weight (calculated based on the ranking of items on the Likert scale).

#### Round 3

In Round 3 we included the items from the top 15 of the male responses and from the top 15 of the female responses.

First, we asked participants if they agreed with the way we combined the items that, in our opinion, reflected the same or very similar content.

Second, we asked patients to select 5 items of the randomly presented list that they considered most crucial in the evaluation of the effect of acute headache medication. We asked patients to value these 5 items by distributing 10 points over these items, such that the item they considered most important was given the highest number of points. Third, we asked participants to indicate how quickly (time to onset) they would want the effect to occur (but only for the symptoms they had experienced themselves).

## Results

### Participants

**Figure 2:**
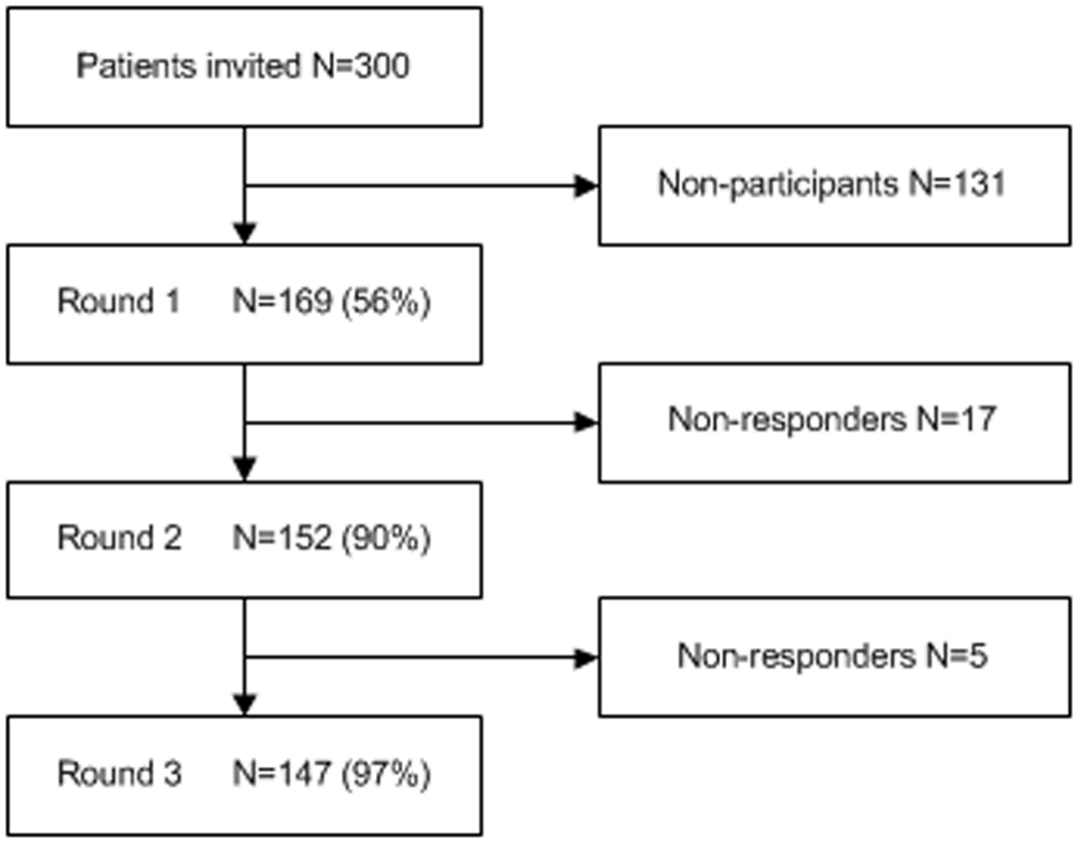
Flowchart of panel member participation.

Figure 2 presents the flow of participants through the study. Of the 300 patients, the first questionnaire (Round 1) was returned by 169 (56%) patients. Participants and non-participants were compared on the following characteristics available from the LUMINA database: age, educational level, headache subtype, headache frequency, medication use, educational level, anxiety scores, and depression scores (data not shown). Of the 169 participants in Round 1, 55% were women (n = 93) and 45% were men (n = 76). There were no significant differences between participants and non-participants on any of the other characteristics.

Response rates (as percentage of the respondents in the previous round) in the consecutive rounds were high, i.e. 90% in Round 2 and 97% in Round 3.

### Round 1

#### Baseline characteristics

The baseline characteristics of participants derived from the first Delphi questionnaire are shown in Table 1.

**Table 1:** Characteristics of panel members (N = 169).

Characteristics	
% female	55.0%
Age in years, median (IQR)	47 (40–57)
Years of fulltime education	13.8 (3.6)
HADS total score	11.0 (6.4)
% ≥1–4 attacks per month	57.4%
% ≥1–4 days per month	74.6%
Number of headache days per month	9.2 (7.8)
Number of migraine headache days per month	7.1 (6.3)
% treatment by general practitioner	48%
% treatment by neurologist	33%
Use of a simple analgesic*, days per month	5.37 (6.4)
Use of ergotamine per month, days per month	0.04 (0.33)
Use of triptan per month, days per month	5.3 (5.4)
Use of medication per month, days per month	6.9 (5.5)
% use of prophylaxis	37%

Values are means (SD) unless stated otherwise.

HADS = Hospital Anxiety and Depression Scale.

* Paracetamol, NSAID, or saridon.

#### First Delphi question: Most bothersome aspects of having migraine attacks

The most frequently mentioned bothersome aspects of having a migraine attack were headache pain (62%) and the impact of migraine headaches on daily life (53%). Answers to this question were similar for male and female respondents. An overview of all answers can be found in Table S1.

#### Second Delphi question: Patients' wishes concerning the effect of attack medication

The effects most frequently wished for were that the medication would take away the headache (18%) and that the medication would have no adverse effects (15%). Although we were investigating the effect that patients wished their attack medication to have, some participants spontaneously mentioned that they thought it was important that the cause of their migraine was treated (5^th^ and 6^th^ place in ranking order) and that the medication had no negative effects on the long term (6^th^ and 7^th^ place in ranking order). These items focused on migraine-related aspects rather than on the direct effect of migraine on the patient. Accordingly, these two items were included in the second round to give patients the opportunity to indicate how important they rated these particular aspects. However, after the second round these types of items were excluded, because our final aim was to compile a short list of outcome measures for migraine research.

#### Evaluation of currently used outcome measures

The ranking of the currently used outcome measures is presented in Table S2. Patients considered the following outcome measures to be the most important for them: decrease of headache, time to effect, no relapse within one day, reliability of medication, and how soon they are able to resume normal activities.

### Round 2

#### Second Delphi question: Patients' wishes concerning the effect of attack medication

After the answers to the second Delphi question were grouped into 36 categories we presented them to the patient panel again and asked to choose the five most important items and evaluate these on a 5-point Likert scale (from 1 = not important, to 5 = very important). The results and the ranking of the items based on can be found in Table S3. The highest ranked items were ‘take away the headache’ and ‘prevent the attack from carrying on’.

As mentioned before, after Round 2 we excluded the items that items focused on migraine-related aspects rather than on the direct effect of migraine on the patient. The excluded items were: ‘Have no or fewer side-effects’, ‘Have no negative effects in the long term’, ‘Treat the cause’, ‘Work as effectively each time’, ‘Not be too expensive’, and ‘Is easy to take in’. Also, excluded was the item ‘Work fast’ as information on the time to effect (speed of onset) was addressed in a separate question.

Of the candidate items, four pairs resembled each other to a considerable extent and were therefore combined. For example, we combined the answers ‘I want the medicine to clear my head’ and ‘I want the medicine to enable me to think clearly again’ into ‘I want the medicine to enable me to think clearly again’. In the third round we explicitly asked participants if they agreed with our decisions concerning the way these items were combined.

### Round 3

#### Combination of items

More than 60% of participants agreed with our combination of the four pairs of similar items.

#### Second Delphi question: Patients' wishes concerning the effect of attack medication

The final results of the Round 3 are presented in Table 2. The items considered most important were: take away the headache, prevent the attack from carrying on, no relapse within one day, and let the patient function properly again.

**Table 2:** Final results of second Delphi question (third round).

Ranking	Outcome measure	N	Mean item weight (SD)	Frequency-weight product*
1	take away the headache	121	3.36 (1.52)	407
2	prevent the attack from carrying through	100	2.55 (1.38)	255
3	make sure no other attack follows within a few hours or within a day	83	1.90 (0.96)	158
4	let me function properly again	83	1.64 (1.04)	136
5	clear my head	56	1.41 (0.11)	79
6	take away the pressing or thumping feeling	43	1.61 (0.19)	69
7	take away the nausea	49	1.35 (0.13)	66
8	take away the problems with vision (light flashes, hazy vision, double vision)	30	2.17 (0.25)	65
9	take away the sense of illness *during a headache attack*	41	1.41 (0.16)	58
10	take away the neck pain	35	1.57 (1.18)	55
11	take away the tiredness	44	1.00 (0.11)	44
12	take way the loss of function (problems with speech, tingling or loss of power in arms/legs)	23	1.70 (0.25)	39
13	take away the persistent headache *after the headache attack*	27	1.19 (0.16)	32

Items considered most important by the participants (N = 147).

* Weight frequency product: weight multiplied by the number of times it is mentioned.

The ranking order for the five highest ranked items did not differ between male and female participants (data not shown). Female participants ranked nausea higher compared to male participants (8^th^ and 15^th^ place in ranking order, respectively). Male participants ranked problems with vision higher compared to female participants (9^th^ and 13^th^ place in ranking order, respectively). These differences are related to a difference in the incidence of these symptoms between male and female participants (i.e. 25.0% of females always experiences nausea, compared to 15.0% of men; 17.5% of females always experience problems with vision compared to 26.9% of men).

The ranking order of the five highest ranked items did not differ between patients who were treated by a neurologist and those not treated by a neurologist (data not shown). Differences lower in the ranking order were also related to a difference in the incidence of symptoms between these two groups.

#### Time to effect

The results of the question on speed of onset are presented in Table 3. According to the respondents, the headache pain, the pressing or thumping feeling, and the accompanying symptoms should have disappeared within 30 min. They accepted a slightly longer induction time of 1 h, for being able to function properly and being able to think clearly again, not feeling lethargic and tired, and being cured of their neck ache.

**Table 3:** Wishes of patients concerning time to effect (third round).

Symptoms		Cumulative percentage of patients					
	N	<15 min	<30 min	<1 h	<2 hours	<3 hours	<1 day
Take away the headache	147	32.0	72.1	94.6	98.6	100	100
Make sure I can function properly again	144	12.5	43.1	77.8	89.6	94.4	100
Take away the pressing or thumping feeling	137	27.0	59.1	90.5	95.6	98.5	100
Take away the sense of illness	138	8.7	34.8	76.1	87.0	92.8	100
Take away the problems with vision (light flashes, hazy vision, double vision)	105	33.3	55.2	83.8	90.5	97.1	100
Take away the nausea	132	40.9	61.4	92.4	95.5	99.2	100
Make sure I can think clearly again	137	13.1	39.4	78.1	90.5	96.4	100
Take away the neck pain	112	14.3	33.9	72.3	84.8	91.1	100
Take away the loss of function (problems with speech, tingling or loss of power in arms/legs)	94	31.9	50.0	74.5	86.2	93.6	100
Take away the tiredness	143	7.0	23.8	49.7	67.1	79.0	100

Each symptom rated only by patients who reported that they experienced this symptom themselves.

## Discussion

### Main results

This Delphi study shows that the outcome measure ‘pain free within 2 hours’ on its own does not sufficiently reflect what is important to migraine patients. Patients want their attack medication to relieve the headache within 30 min, rather than the currently used criterion ‘pain free within two hours’. They also want the medication to prevent the attack from carrying on, to prevent recurrence, and allow them to function properly within 1 h. This applies to both male and female patients, and to patients treated by a neurologist and not treated by a neurologist.

### Strengths and weaknesses

This is the first study in which migraine patients were specifically asked what they consider important with respect to the development of new attack medication for migraine, in a setting where they were not influenced by their fellow patients and/or an interviewer. This allowed them to freely form and express their personal opinions. Also, the Delphi design enabled us to start with an explorative open-ended question in the first round and, subsequently, to ask patients to evaluate the answers that were given and specify the desired ‘time to effect’ in the second and third rounds. We consider this a major and distinctive strength and of the present study. In addition, the present study is representative for patients from both general practice and secondary/tertiary care.

The study also has some limitations. First, the population might be somewhat higher educated than the migraine population in general, as they had to fill in questionnaires via internet. These patients might have more insight about migraine and headaches, which could have influenced the results. Also, patients who respond well to their current medication and had less headaches might have answered questions differently from patients who did not respond well to their medication or had severe headaches. Secondly, inherent to the study is that subjective choices had to be made when formulating items and constructing the questionnaires. However, this was carefully performed by i) involving a health psychologist with no background in migraine research as to enable more objective decision-making, ii) categorising the answers to the open-ended questions independently, and iii) requiring consensus from all authors when designing the questionnaires. Thirdly, the Delphi procedure is a consensus method in which opinions of individual participants that are not supported by others will not come up in the final result. In order to ensure that no valuable opinion that was supported by many participants would be missed, we fed back all opinions that were expressed in Round 1 to the participants in Round 2. In this way, all patients had the possibility to reflect on the suggestions of co-participants which they had not thought of themselves.

### Comparison literature

A telephone survey of migraine patients among the general population showed that migraine patients rate the following items as the most important attributes of acute migraine treatment: complete relief of head pain, lack of recurrence and rapid onset of pain relief. In that survey 71% of the patients wanted the pain to be gone in less than 30 min [12]. After existing outcome measure had been presented to our patients, this Delphi study allowed them to suggest medication effects that they considered to be important; this yielded two new items, i.e. ‘Prevention of worsening of the attack’ and ‘The ability to function properly again within 1 hour’.

It is reported that most migraine patients (54%) do not notice any benefit in the first hour after taking headache medication [13]. Remarkably, although the wish for a faster effect of attack medication was already expressed by patients in a study published in 1999 [12], the outcome measures used in the evaluation of medication have not yet been altered.

## Conclusions

The currently used outcome measures in migraine research do not sufficiently reflect the expectations of migraine patients. The present study shows that patients wish their headache to be taken away within 30 min. It seems that, until now, research on migraine medication has been guided by what was considered possible and not by the actual wishes/expectations of migraine patients. The results of the present study clearly indicate that treatment should focus on: being pain free rapidly, preventing the migraine from becoming worse, preventing the recurrence of migraine, restoring proper function and permitting patients to think clearly again within an hour. Future research should aim to develop an outcome measure that combines all these aspects and thereby enable measurement of what migraine patients find most important.

## Supporting Information

Table S1 **Categorized answers to the first Delphi question (Round 1): ‘What do you consider to be the most bothersome about having migraine attacks?’** * Chi square test. ^#^ Not otherwise specified, binge eating, disorientation.
(DOC)

Table S2 **Evaluation of current outcome measures ranked in order of importance (first round).** ^*^ Number of patients who experience this symptom during their migraine attacks.
(DOC)

Table S3 **Ranking of the 36 items in the second Delphi round for female and male respondents (Round 2).**
(DOC)

Appendix S1 **Delphi questionnaires round 1, 2, and 3.**
(DOC)
