# Supplementary material for: What Do Patients Consider to Be the Most Important Outcomes for Effectiveness Studies on Migraine Treatment? Results of a Delphi Study
Source: PLoS One. 2014 Jun 16;9(6):e98933. doi: 10.1371/journal.pone.0098933 (PMC4059644; doi:10.1371/journal.pone.0098933)
Supplement: Table S1 — Categorized answers to the first Delphi question (Round 1): ‘What do you consider to be the most bothersome about having migraine attacks?’ * Chi square test. # Not otherwise specified, binge eating, disorientation. (DOC) [file pone.0098933.s001.doc]

Table S1. Categorized answers to the first Delphi question (Round 1): ‘What do you consider to be the most bothersome about having migraine attacks?’

|  | **Men (%)**  **(n=76)** | **Women (%)**  **(n=93)** | **P-value*** | **Total** |
| --- | --- | --- | --- | --- |
| Headache | 42 (55.3) | 62 (66.7) | 0.13 | 104 |
| Not being able to function normally and the impact on social life/work/family | 44 (57.9) | 55 (59.1) | 0.87 | 98 |
| Nausea/vomiting, less appetite during attack | 30 (39.5) | 45 (48.4) | 0.25 | 75 |
| Hypersensitivity to light, sound, smell | 12 (15.8) | 30 (32.3) | 0.01 | 42 |
| Tiredness, yawning | 13 (17.1) | 22 (23.7) | 0.30 | 35 |
| Problems with concentration, sense of absence | 17 (22.4) | 17 (18.3) | 0.51 | 34 |
| Visual (aura) phenomena | 14 (18.4) | 12 (12.9) | 0.32 | 26 |
| Psychological consequences (anxiety, gloom) | 13 (17.1) | 13 (14.0) | 0.58 | 26 |
| Irritability/moodiness | 8 (10.5) | 13 (14.0) | 0.50 | 21 |
| Recovery period after the attack (tiredness, concentration problems, sense of illness) | 6 (7.9) | 10 (10.8) | 0.53 | 16 |
| The need to take medication | 4 (5.30 | 10 (10.8) | 0.20 | 14 |
| General sense of illness | 4 (5.3) | 9 (9.7) | 0.28 | 13 |
| Neurological loss of function (not being able to speak properly, not being able to come to words, loss of function in the face/limbs) | 4 (5.3) | 8 (8.6) | 0.40 | 12 |
| Unpredictability of attacks | 5 (6.6) | 4 (4.3) | 0.51 | 10 |
| That it lasts several days | 4 (5.3) | 6 (6.5) | 0.75 | 10 |
| Aura (not specified) | 7 (9.2) | 2 (2.2) | 0.42 | 9 |
| Sleeping problems (sleeping badly, sleeping a lot) | 6 (7.9) | 3 (3.2) | 0.18 | 9 |
| Misunderstanding from the surroundings | 4 (5.30 | 2 (2.2) | 0.28 | 6 |
| Neck pain | 2 (2.6) | 6 (5.6) | 0.25 | 8 |
| Dizziness | 4 (5.3) | 1 (1.1) | 0.11 | 5 |
| Phenomena prior to attack^#^ | 0 (0.0) | 3 (3.2) | 0.11 | 3 |
| Changed sense of taste | 0 (0.0) | 2 (2.2) | 0.20 | 2 |
| Pain to touch (face, ears) | 0 (0.0) | 2 (2.2) | 0.20 | 2 |
| *Total* | *243* | *335* |  | *578* |

***** Chi square test

^#^ Not otherwise specified, binge eating, disorientation
