# Supplementary material for: What Do Patients Consider to Be the Most Important Outcomes for Effectiveness Studies on Migraine Treatment? Results of a Delphi Study
Source: PLoS One. 2014 Jun 16;9(6):e98933. doi: 10.1371/journal.pone.0098933 (PMC4059644; doi:10.1371/journal.pone.0098933)
Supplement: Table S2 — Evaluation of current outcome measures ranked in order of importance (first round). * Number of patients who experience this symptom during their migraine attacks. (DOC) [file pone.0098933.s002.doc]

Table S2. Evaluation of current outcome measures ranked in order of importance (first round).

| **Outcome measure** | **n^*^** | **% of total** | **Mean (SD)** |
| --- | --- | --- | --- |
| Decrease of headache | 168 | 99.4 | 4.9 (0.4) |
| Time to effect | 169 | 100 | 4.6 (0.7) |
| Relapse within one day | 165 | 98.0 | 4.5 (0.8) |
| Reliability of medication | 168 | 99.4 | 4.5 (0.7) |
| How soon able to resume normal activities | 167 | 98.8 | 4.5 (0.8) |
| How soon feeling completely well | 169 | 100 | 4.4 (0.9) |
| How soon being able to think clearly | 163 | 96.4 | 4.3 (1.0) |
| Decrease of nausea | 139 | 82.2 | 4.1 (1.0) |
| Adverse events | 162 | 95.9 | 4.1 (1.0) |
| If more than 1 dose needed | 168 | 99.4 | 3.9 (1.3) |
| Decrease of photophobia | 141 | 83.4 | 3.7 (1.4) |
| Decrease of shoulder and/or neck pain | 133 | 78.7 | 3.7 (1.3) |
| Decrease of irritability | 149 | 88.2 | 3.7 (1.2) |
| Decrease of phonophobia | 147 | 87.0 | 3.6 (1.2) |

**^*^** Number of patients who experience this symptom during their migraine attacks.
