# Supplementary material for: What Do Patients Consider to Be the Most Important Outcomes for Effectiveness Studies on Migraine Treatment? Results of a Delphi Study
Source: PLoS One. 2014 Jun 16;9(6):e98933. doi: 10.1371/journal.pone.0098933 (PMC4059644; doi:10.1371/journal.pone.0098933)
Supplement: Table S3 — Ranking of the 36 items in the second Delphi round for female and male respondents (Round 2). (DOC) [file pone.0098933.s003.doc]

Table S3. Ranking of the 36 items in the second Delphi round for female and male respondents (Round 2).

|  | **Female respondents** | **N** | **Mean item**  **weight (SD)** | **Male respondents** | **N** | **Mean item**  **weight (SD)** |
| --- | --- | --- | --- | --- | --- | --- |
| **1** | take away the headache | 63 | 4.98 (0.16) | take away the headache | 50 | 4.92 (0.27) |
| **2** | prevent the attack from carrying on | 45 | 4.96 (0.21) | prevent the attack from carrying on | 31 | 5.00 (0.00) |
| **3** | make sure no other attack follows within a few hours or within one day | 38 | 4.97 (0.16) | let me function properly again | 26 | 4.54 (0.76) |
| **4** | let me function properly again | 29 | 4.86 (0.44) | make sure no other attack follows within a few hours or within one day | 22 | 4.95 (0.21) |
| **5** | take away the pressing or thumping feeling | 25 | 4.92 (0.28) | have no negative effects on the long term | 20 | 4.95 (0.22) |
| **6** | have no negative effects on the long term | 20 | 5.00 (0.00) | treat the cause | 20 | 4.45 (1.05) |
| **7** | treat the cause | 20 | 4.40 (0.99) | work fast | 17 | 4.41 (1.06) |
| **8** | work fast | 17 | 4.82 (0.53) | take away the pressing or thumping feeling | 15 | 4.87 (0.35) |
| **9** | take away the nausea | 15 | 5.00 (0.00) | take away the sense of illness *during a headache attack* | 13 | 4.69 (0.48) |
| **10** | work as effectively each time | 12 | 4.75 (0.45) | make sure I can think clearly again | 11 | 4.73 (0.65) |
| **11** | take away the problems with vision *prior to a headache attack* (light flashes, hazy vision, double vision) | 12 | 4.67 (0.65) | take away the problems with vision *prior to a headache attack* (light flashes, hazy vision, double vision) | 11 | 4.64 (0.81) |
| **12** | have no or fewer side-effects | 12 | 4.58 (1.16) | clear my head | 9 | 4.78 (0.44) |
| **13** | take away the persistent headache *after the headache attack* | 12 | 4.50 (0.52) | prevent me from having to throw up | 9 | 4.56 (1.01) |
| **14** | take way the loss of function (problems with speech, tingling or loss of power in arms/legs) | 11 | 4.82 (0.40) | work as effectively each time | 8 | 5.00 (0.00) |
| **15** | take away the tiredness *after a headache attack* | 11 | 4.55 (0.69) | take away the problems with vision *during the headache attack* (light flashes, hazy vision, double vision) | 8 | 4.63 (0.52) |
| **16** | take away the neck pain | 10 | 4.90 (0.32) | take away the persistent headache *after the headache attack* | 7 | 4.86 (0.38) |
| **17** | take away the sense of illness *during a headache attack* | 11 | 4.45 (0.69) | take away the tiredness *during a headache attack* | 7 | 4.71 (0.49) |
| **18** | prevent me from having to throw up | 10 | 4.70 (0.67) | let me be able to sleep | 7 | 4.57 (0.79) |
| **19** | make sure I can think clearly again | 9 | 4.56 (0.53) | take away the neck pain | 7 | 4.43 (0.79) |
| **20** | take away the sensitivity to outside stimulants (light, noise, or smells) | 7 | 4.29 (0.76) | take away the nausea | 7 | 4.29 (0.79) |
| **21** | take away the problems with vision *during the headache attack* (light flashes, hazy vision, double vision) | 6 | 4.83 (0.41) | let me be able to relax | 5 | 4.80 (0.45) |
| **22** | let me be able to sleep | 6 | 4.67 (0.52) | take way the loss of function (problems with speech, tingling or loss of power in arms/legs) | 5 | 4.60 (0.89) |
| **23** | be easy to swallow/take in | 5 | 5.00 (0.00) | take away the tiredness *after a headache attack* | 5 | 4.60 (0.89) |
| **24** | take away the tiredness *during a headache attack* | 4 | 4.50 (0.58) | have no or fewer side-effects | 5 | 4.60 (0.55) |
| **25** | clear my head | 3 | 4.67 (0.58) | take away the sensitivity to outside stimulants (light, noise, or smells) | 4 | 4.75 (0.50) |
| **26** | take away the sense of illness *after a headache attack* | 3 | 4.67 (0.58) | take away the shoulder pain | 3 | 4.67 (0.58) |
| **27** | take away the other preceding phenomena (such as (binge)eating, yawning) | 1 | 5.00 (-) | take away the bowel complaints | 3 | 4.67 (0.58) |
| **28** | take away the irritability or moodiness *during a headache attack* | 1 | 5.00 (-) | take away the irritability or moodiness *prior to a headache attack* | 3 | 4.33 (1.15) |
| **29** | take away the bowel complaints | 1 | 5.00 (-) | be easy to swallow/take in | 3 | 3.67 (2.31) |
| **30** | take away the dizziness *during a headache attack* | 1 | 5.00 (-) | take away the dizziness *during a headache attack* | 2 | 4.50 (0.71) |
| **31** | let me be able to relax | 1 | 5.00 (-) | take away the other preceding phenomena (such as (binge)eating, yawning) | 2 | 3.00 (2.83) |
| **32** | make sure my sense of taste is normal again | 1 | 4.00 (-) | not be too expensive | 1 | 4.00 (-) |
| **33** | take away the irritability or moodiness *prior to a headache attack* | 1 | 1.00 (-) | take away the sense of illness *after a headache attack* | 1 | 3.00 (-) |
| **34** | take away the shoulder pain | 0 | - | make sure my sense of taste is normal again | 0 | - |
| **35** | make sure I no longer have a sensitive skin | 0 | - | take away the irritability or moodiness *during a headache attack* | 0 | - |
| **36** | not be too expensive | 0 | - | make sure I no longer have a sensitive skin | 0 | - |
