# Supplementary material for: What Do Patients Consider to Be the Most Important Outcomes for Effectiveness Studies on Migraine Treatment? Results of a Delphi Study
Source: PLoS One. 2014 Jun 16;9(6):e98933. doi: 10.1371/journal.pone.0098933 (PMC4059644; doi:10.1371/journal.pone.0098933)
Supplement: Appendix S1 — Delphi questionnaires round 1, 2, and 3. (DOC) [file pone.0098933.s004.doc]

Appendix S1. Delphi questionnaires round 1, 2, and 3

**Questionnaire round 1**

1. How many severe headache attacks have you experienced in the past 12 months?

🞏 1 - 2

🞏 3 - 6 (i.e. on average one attack per 2 - 4 months)

🞏 7 - 12 (i.e. on average one attack per 1 - 2 months)

🞏 13 - 54 (i.e. on average one to four attacks per month)

🞏 more than 54 (i.e. multiple attacks per week)

🞏 multiple attacks per day

🞏 I had a headache almost continuously without increased paroxysm

2. On how many days in the past 12 months did you experience severe headaches?

🞏 1 - 2

🞏 3 - 6 (i.e. on average one day per 2 - 4 months)

🞏 7 - 12 (i.e. on average one day per 1 - 2 months)

🞏 13 - 54 (i.e. on average one to four days per month)

🞏 more than 54 (i.e. multiple days per week)

🞏 almost every day

3. On how many days per month did you experience headaches in the past 3 months?

[………] days per month

4. On how many of those days did you experience severe paroxysmal headache?

[………] days per month

**5. Preventive medication (to prevent occurrence of headache)**

The following question is about preventive medication that was prescribed to you by a medical doctor to prevent headache complaints (e.g. propanolol (Inderal), metoprolol (Selokeen, Lopresor), natriumvalproate (Depakine), topiramate (Topamax), flunarizine (Sibelium), pizotifeen (Sandomigran)).

5A. Are you taking daily prophylactic medication prescribed to you by a medical doctor to prevent your complaints?

🞏 no *[🡺 go directly to question 6]*

🞏 yes

*You only need to fill in this question if 5A=yes*

5B. Please write down the name of your daily preventive medication.

[………………………………]

**6. Medication to be taken during an attack**

The following questions are supplementary questions about your medication use. We ask about the number of days that you use certain medication against headache complaints. We ask you to take the mean use of the last three months. If you take less than 1 dose per month, we ask you to fill in a 0. Also, if you take a medication (almost) never, we ask you to fill in a 0.

6A. On how many days in the past 3 months did you use simple analgesics, such as paracetamol, aspirin, naproxen, diclofenac, ibuprofen, saridon etc.?

[………] days per month

6B. On how many days in the past 3 months did you use ergotamines, such as cafergot, ergocaffeine?

[………] days per month

6C. On how many days in the past 3 months did you use triptans, such assumatriptan (Imigran), eletriptan (Relpax), rizatriptan (Maxalt), almotriptan (Almirall), zolmitriptan (Zomig), frovotriptan (Fromirex) of naratriptan (Naramig))?

[………] days per month

6D. Possibly there are days on which you take several kinds of medication. On how many days per month do you take any kind of medication against your headache?

[………] days per month

**7. Treatment**

7A. Are you currently being treated by a general practitioner for your migraine?

🞏 no

🞏 yes

7B. Are you currently being treated by a neurologist for your migraine?

🞏 no

🞏 yes

People perceive different things as bothersome when having a migraine attack. We would like to know what things you find bothersome about having a migraine attack.

8. What do you find most bothersome about having a migraine attack? Please mention at least 3 and maximum 5 things that you find bothersome about having a migraine attack. Please indicate how bothersome you find these things on a 5-point scale.

| Things that are bothersome about having a migraine attack | How bothersome?  1 = not at all bothersome  5 = very bothersome | Additional remarks  (If you wish, you can add an additional remark) |
| --- | --- | --- |
|  | **1 2 3 4 5** |  |
|  | **1 2 3 4 5** |  |
|  | **1 2 3 4 5** |  |
|  | **1 2 3 4 5** |  |
|  | **1 2 3 4 5** |  |

9. If a new medicine was to be developed against migraine attacks, what would you wish the effect to be? Please mention at least 3 and maximum 5 things that you find important as a result of attack medication for migraine. Please indicate how important you consider these things.

People have different expectations from migraine medication. We would like to know which complaints you want to be treated.

| What do you expect from migraine medication? | How important?  1 = not important  5 = very important | Additional remarks  (If you wish, you can add an additional remark) |
| --- | --- | --- |
|  | **1 2 3 4 5** |  |
|  | **1 2 3 4 5** |  |
|  | **1 2 3 4 5** |  |
|  | **1 2 3 4 5** |  |
|  | **1 2 3 4 5** |  |

10. You have indicated what you considered important in the treatment of your migraine attacks. Below is an overview of aspects in which research is being done about new medication. We would like to know how you consider these. Do you consider it to be important, less important or not important? Could you indicate, per subject, how important you consider it to be? If the subject is not applicable to you (e.g. because you have never suffered from it), you may indicate ‘not applicable to me’. Finally, there is some space to add additional remarks that you think are missing from the list.

| Subject | How important?  1 = not very important  5 = very important | Not applicable to me | Additional remarks  (If you wish, you can add an additional remark) |
| --- | --- | --- | --- |
| Decrease of the headache | **1 2 3 4 5** |  |  |
| Decrease of nausea | **1 2 3 4 5** |  |  |
| Decrease of light sensitivity | **1 2 3 4 5** |  |  |
| Decrease of noise sensitivity | **1 2 3 4 5** |  |  |
| Decrease in neck/shoulder pain | **1 2 3 4 5** |  |  |
| Decrease in irritability | **1 2 3 4 5** |  |  |
| Rate at which you can think clearly again after taking medication | **1 2 3 4 5** |  |  |
| Is one tablet/suppository/injection/spray sufficient? | **1 2 3 4 5** |  |  |
| How quickly you can resume your activities | **1 2 3 4 5** |  |  |
| How quickly you feel completely recovered | **1 2 3 4 5** |  |  |
| The length of time for the medication to take effect | **1 2 3 4 5** |  |  |
| Do complaints return within one day? | **1 2 3 4 5** |  |  |
| Does the medication as effectively with each attack? | **1 2 3 4 5** |  |  |
| Do you suffer from side-effects? | **1 2 3 4 5** |  |  |

**Additional remarks about the questionnaire**

Please write down below any comments about the questionnaire

Thank you very much for your co-operation!

**Questionnaire round 2**

In the previous questionnaire we asked you to name aspects that were most important to you in the treatment of a migraine attack. Below, all the answers are presented.

Part A

Could you indicate per answer how important this aspect is to you on a scale of 1 (not important) to 5 (very important)?

The medication must:

1. take away the irritability or moodiness *prior to a headache attack*
2. take away the other preceding phenomena (such as (binge)eating, yawning)
3. prevent the attack from carrying on
4. take away the problems with vision *prior to a headache attack* (light flashes, hazy vision, double vision)
5. take way the loss of function (problems with speech, tingling or loss of power in arms/legs)
6. take away the headache
7. take away the pressing or thumping feeling
8. take away the nausea
9. prevent me from having to throw up
10. take away the neck pain
11. take away the shoulder pain
12. take away the problems with vision *during the headache attack* (light flashes, hazy vision, double vision)
13. clear my head
14. make sure I can think clearly again
15. take away the sensitivity to outside stimulants (light, noise, or smells)
16. make sure my sense of taste is normal again
17. take away the irritability or moodiness *during a headache attack*
18. take away the sense of illness *during a headache attack*
19. take away the tiredness *during a headache attack*
20. let me function properly again
21. that I no longer have sensitive skin
22. take away the bowel complaints
23. take away the dizziness *during a headache attack*
24. let me be able to relax
25. let me be able to sleep
26. take away the persistent headache *after the headache attack*
27. take away the tiredness *after a headache attack*
28. take away the sense of illness *after a headache attack*
29. make sure no other attack follows within a few hours or within one day
30. work fast
31. have no or fewer side-effects
32. have no negative effects in the long term
33. treat the cause
34. work as well each time
35. not be too expensive
36. is easy to swallow/take in

Part B

Of the above aspects, which 5 do you consider the most important and must definitely be included in the development of new medication against migraine attacks?

Could you rank what you consider to be the most important aspect as number 1, the next most important as number 2, etc.

🞏 must take away the irritability or moodiness *prior to a headache attack*

🞏 take away the other preceding phenomena (such as (binge)eating, yawning)

🞏 prevent the attack from carrying on

🞏 take away the problems with vision *prior to a headache attack* (light flashes, hazy vision, double vision)

🞏 take away the loss of function (problems with speech, tingling or loss of power in arms/legs)

🞏 take away the headache

🞏 take away the pressing or thumping feeling

🞏 take away the nausea

🞏 prevent me from having to throw up

🞏 take away the neck pain

🞏 take away the shoulder pain

🞏 take away the problems with vision *during the headache attack* (light flashes, hazy vision, double vision)

🞏 clear my head

🞏 make sure I can think clearly again

🞏 take away the sensitivity to outside stimulants (light, noise, or smells)

🞏 make sure my sense of taste is normal again

🞏 take away the irritability or moodiness *during a headache attack*

🞏 take away the sense of illness *during a headache attack*

🞏 take away the tiredness *during a headache attack*

🞏 let me function properly again

🞏 that I no longer have sensitive skin

🞏 take away the bowel complaints

🞏 take away the dizziness *during a headache attack*

🞏 let me be able to relax

🞏 let me be able to sleep

🞏 take away the persistent headache *after the headache attack*

🞏 take away the tiredness *after a headache attack*

🞏 take away the sense of illness *after a headache attack*

🞏 make sure no other attack follows within a few hours or within one day

🞏 work fast

🞏 have no or fewer side-effects

🞏 have no negative effects on the long term

🞏 treat the cause

🞏 work as effectively each time

🞏 not be too expensive

🞏 is easy to swallow/take in

Could you indicate below which aspects you still miss and that, in your opinion, should definitely be included in the review of the efficacy of the new medication?

**Questionnaire round 3**

Part A

As a result of the answers to the previous questions we have combined some of the questions. We would like to know whether, in your opinion, you feel that these questions can be combined. It might be that you would give a different answer to the individual questions and that, therefore, they individual questions should not be combined.

Below, each time, we present two questions from the previous round with our proposal as how to they can be combined. We would like to know whether you agree with the way we have combined them.

Answering options:

- Agree: these two items can be combined
- Disagree: these two items should remain separated, because they ask about different aspects

1.

Questions from the previous round:

- take away the problems with vision *prior to the headache attack* (light flashes, hazy

vision, double vision)

- take away the problems with vision *during the headache attack* (light flashes, hazy vision, double vision)

Combined to:

- take away the problems with vision prior to or during the headache attack (light flashes, hazy vision, double vision).

2.

Questions from the previous round:

- clear my head again
- make sure I can concentrate again

Combined to:

- make sure I can think clearly again

3.

Questions from the previous round:

- take away the nausea
- make sure I do not have to vomit

Combined to:

- take away the nausea

4.

Questions from the previous round:

- take away the tiredness *after the headache attack*
- take away the tiredness *during the headache attack*

Combined to:

- take away the tiredness during or after the headache attack

Part B

Below, we list the items from the previous round that were indicated to be the most important aspects regarding the effect which new migraine medication should have.

From the list, please choose 5 aspects that are the most important to you.

To have an idea of how important you consider the various aspects, we ask you to distribute 10 points over the 5 aspects you have chosen, based on their level of importance. Thus, an important item will be given more points than a less important item.

Examples:

1. You experience aspect 1 as very important, whereas you do not find the other aspects to be important at all.
   In this case, you fill in:
   1. Effect 1 10 points
   2. Effect 6 0 points
   3. Effect 2 0 points
   4. Effect 3 0 points
   5. Effect 4 0 points
2. You experience aspect 1 and aspect 6 to be the most important. In addition, you consider aspect 2 to be fairly as important and aspects 3 and 4 to be only slightly important. In this case, you fill in:
3. Effect 1 3 points
4. Effect 6 3 points
5. Effect 2 2 points
6. Effect 3 1 point
7. Effect 4 1 point

The medication must …

1. take away the headache
2. prevent the attack from carrying on
3. make sure no other attack follows within a few hours or within one day
4. let me function properly again
5. take away the pressing or thumping feeling
6. take away the sense of illness *during a headache attack*
7. take away the problems with vision (light flashes, hazy vision, double vision)
8. take away the nausea
9. make sure I can think clearly again
10. take away the persistent headache *after the headache attack*
11. take away the neck pain
12. take way the loss of function (problems with speech, tingling or loss of power in arms/legs)
13. take away the tiredness

Part C

For some of the aspects, we would like to know whether you still suffer, or have suffered from these items with your migraine attacks.

Answering options:

1. I never have this
2. I sometimes have this
3. I often have this
4. I always have this
5. headache
6. pressing or thumping feeling
7. sense of illness
8. problems with vision (light flashes, hazy vision, double vision)
9. nausea
10. not being able to think clearly
11. persistent headache *after the headache attack*
12. neck pain
13. loss of function (problems with speech, tingling or loss of power in arms/legs)
14. tiredness

Part D

In the previous list of questions many people indicated that the speed at which the medication acts is important. We would like to know from you, how fast you would like the medication to act on particular symptoms.

How fast should the following effects of the medication take place?

Answering options:

1. within fifteen minutes
2. within thirty minutes
3. within 1 h
4. within two hours
5. within three hours
6. within one day
7. I never suffer from this symptom
8. take away the headache
9. make sure I can function properly again
10. take away the pressing or thumping feeling
11. take away the sense of illness *during a headache attack*
12. take away the problems with vision (light flashes, hazy vision, double vision)
13. take away the nausea
14. make sure I can think clearly again
15. take away the neck pain
16. take away the loss of function (problems with speech, tingling or loss of power in arms/legs)
17. take away the tiredness
